# The Impact of Music Therapists’ Perspectives on Quality of Life in Building Relationships with Older Adults with Chronic Illness

**DOI:** 10.3390/bs12110439

**Published:** 2022-11-10

**Authors:** Kyung Min Kim

**Affiliations:** Faculty of Music Therapy, Boyer College of Music and Dance, Temple University, Philadelphia, PA 19122, USA; mindykimt@gmail.com

**Keywords:** music therapy, chronic illness, quality of life, older adults, therapeutic relationship

## Abstract

Older adults with chronic illnesses have diminished qualities of life (QoL) due to physical and mental decline. To promote their QoL, music therapists create meaningful shared music experiences and positive therapeutic relationships to address their psychosocial needs. However, within this relationship-building process, healthcare professionals, staff, and even caregivers appear to project their own perceptions and expectations of what positive QoL of older adults should be. This misapprehension may challenge therapists to meet older adults’ actual QoL needs adequately. To date, no studies have explored music therapists’ perspectives on QoL in building relationships with older adults experiencing chronic illness. Eleven music therapists participated in online, semi-structured phenomenological interviews that were thematically analyzed. Three themes emerged: participants gained an increased awareness of unconscious biases, a deepened understanding of older adults’ QoL, and a purposeful alignment with older adults’ QoL. These highlighted the significance of therapists’ consistent self-reflection within the relationship-building process. Identifying their shared commonalities with older adults guided therapists to engage with clients more salutogenically and empathically. Additionally, recognizing their own biases being projected onto older adults empowered therapists to be more intentional to reconcile their perceptual discrepancies while prioritizing older adults’ authentic voices and capable selves.

## 1. Introduction

Older adults (age 65 or older) comprise 9% of the population worldwide and 19% of the combined population in higher-developed countries [1]. According to World Population Aging 2020 Highlights, the global number of 727 million older adults is expected to increase from 9.3% of the worldwide population in 2020 to 16% in 2050 [2]. This phenomenon has resulted in a continuous advancement in science and improvements in medical technology that can now provide better living conditions and more effective preventative strategies for older adults [3]. In addition, Korkmaz et al. [4] explained that older adults’ increased life expectancy has led to increased health expenditures for them [1] as well as a committed interest in improving both older adults’ QoL and the attitudes society has towards them. Despite having ample medical and pharmaceutical support, older adults still inevitably experience many complications in life and one of the most prevalent complications is chronic illness. The condition of chronic illness accounts for more than 50% of the global disease burden [5]. Chronic illness is the leading cause of death worldwide, accounting for 41 million deaths each year, equivalent to 71% of all deaths globally [6]. Having chronic conditions as well as aging are negative determinants of QoL in the aging population [7] In addition to their natural aging, their chronic conditions could accelerate or worsen their declines in multiple areas of their lives which could also deteriorate their daily functioning [8] and hinder them from living to their fullest potential [9] These declines encompass their loss of memory [10], autonomy [4], status and relationship [11,12], social support [13,14,15], and financial income [16]. These losses are closely associated with their age-related circumstances, retirement, widowhood, and diminished social networks [11]. Moreover, their feelings of isolation and loneliness [12] and anonymity and indistinctiveness [16] can amplify the reality of their loss of independence and their inability to continue vocational activities [17,18]. Unfortunately, as these debilitative conditions progress over a prolonged period, the QoL of older adults will predictably diminish.

While QoL is a difficult concept to define or fully understand, it is one of the key determinants that ensures older adults with chronic illness to experience meaningfulness in their lives despite their illness [19]. Their optimal QoL can be characterized as having no negative symptoms or side-effects of medical treatments [17], having continued vocational activities [20], adapting coping strategies [21,22], maintaining the ability to self-care [23], having spiritual and religious beliefs for comfort and purpose in life [18], feeling hopeful and fulfilled [19], and having a sense of companionship through social support from families and friends [17,24]. Bowling et al. [25] suggests that over 80 percent of older adults agreed that the most common component of their positive QoL was good social relationships. Similar studies resonate with this finding that having good social relationships or networks provided older adults with emotional and informational support [14], feelings of capability [15], feelings of being heard and understood [19], and more interactional opportunities [12]. It is important to note that the frequency and quality of these relationships [14,15,26] are regarded as key predictors to a greater QoL of older adults [11]. Dinc and Gastmans [27] also explain that many healthcare professionals consider having an “awareness of patients’ unvoiced needs, reassurance, encouragement, acceptance of patients’ cultures, lifestyles, and decisions without prejudgment” (p. 507) as required for building positive therapeutic relationship. Overall, these psychosocial benefits of positive relationships result in a positive effect on individuals [28] as an ultimate “protector of health” [26] (p. 6). These findings heighten awareness of the complexity of building relationships and the possible exploration of intricate factors involved in the relationship-building processes.

Bruscia [29] describes that music therapy is “a reflective process wherein the music therapist helps the client to optimize the client’s health, using various facets of the music experience and the relationships formed through them as the impetus for change” (p. 36). It is not only that music creates “an encounter with feelings and emotion which forms the basis for the therapeutic relationship” [30] (p. 47), but also that it provides older adults with a sense of well-being and overall health [31]. Music therapists, then, are a guide fostering these positive therapeutic experiences [16]. Furthermore, music therapy has been effectively used with older adults with chronic illness to reduce pain, anxiety, stress, and depressive symptoms [22,32]; to express emotions and feelings [32,33]; to regulate mood [34]; and to have a new awareness and a sense of meaning in life [35]. The psychosocial model of music in dementia demonstrates that meaningful musical experiences provide emotional connectedness, which enhances the therapeutic relationship and strengthens the core of the therapeutic interventions [36]. When the clients experience more positive emotional responses and a deeper level of comfort and openness with the therapist [8], the more confidence their musical expressions become [28,37]. In addition, group music therapy experiences help them regain social and communication skills, and a sense of belonging [38,39]. Thus, this musical and emotional connectedness not only motivates older adults to engage in positive interpersonal and cooperative verbal interactions with therapists [16,40], but also to discover their positive sense of self, agency, and wellness [12], which ultimately results in a better QoL for older adults.

However, when it comes to building therapeutic relationships with older adults experiencing chronic illness, healthcare professionals, staff, or caregivers appear to project their own perceptions and expectations of what the QoL of older adults should be [10]. This creates discrepancies in QoL ratings of older adults where their QoL is often rated by clinicians, family, or friends [41] especially when the older adults are unable to volitionally express their opinions. Holopainen et al. [19] explains that the family members often underestimate the QoL of their loved ones because they only believe what they see in their loved ones’ agitated behaviors; this can cause family members’ perceptions to be inaccurate. In addition, if caregivers are emotionally depressed and burdened, they tend to project their negative biases [42] and their negative feelings about their own QoL [10], which results in having unreliable perceptions of older adults’ QoL. As some QoL measurements are often based on the signs of older adults’ life satisfaction [26] from others’ points of view, it seems appropriate to rightly understand this concept from the point of view of older adults so that the biases about the QoL of older adults can be potentially minimized. Korkmaz et al. [4] proposes that when people have stereotypes of aging and use those in building relationships with older adults, they will most likely overlook older adults’ individual differences and unique circumstances. Unfortunately, these mistaken perceptions may oppress older adults by subtly influencing them to accept others’ biased attitudes and to live based on what others impose on them [16].

Given the significance of positive therapeutic relationships for older adults’ positive QoL, it is important to explore and understand music therapists’ personal perspectives of QoL as these can be greatly influenced by their various personal lived experiences of connecting with older adults experiencing chronic illness. In fact, in building therapeutic relationships with older adults, music therapists can unintentionally impose their unconscious biases about older adults’ positive QoL. To date, there have been no studies examining therapists’ own internal and unconscious perspectives on QoL nor exploring whether these perspectives influence their relationship-building process with older adults experiencing chronic illness. There have been some studies in other disciplines that explored aspects of the therapeutic relationship. For example, a study of mental health nurses found that being authentic toward clients was pivotal to establish a more intimate relationship [43], whereas counseling psychologists regarded the concept of trust to be essential within their relationship-building process [44]. Palmadottir [7] suggests that when occupational therapists impose their personal assumptions, preferences, and needs in building relationships with older adults, their client-therapist relationships may not be as intimate and reciprocal. Additionally, Browne et al. [45] recommend examining the components of the therapeutic relationship in a more reflective and non-strategic manner. Overall, none of these disciplines appear to examine therapists’ internal and unconscious processes that occur within their relationship-building process. Thus, the purpose of this study is to gain insight into music therapists’ relationship-building processes with older adults with chronic illness by exploring music therapists’ perspectives on QoL, music, and the therapeutic relationship.

### Research Question

How do music therapists experience their own perspectives on QoL and its influence on their relationship-building process with older adults with chronic illness?

Sub question: How do music therapists perceive the role of music in the relationship-building process with older adults with chronic illness?

## 2. Methods

### 2.1. Design

This study employed a hermeneutic phenomenology [46] using conversational interviews, which allowed the researcher to engage with the participants’ lived experiences phenomenologically and interpret the underlying meaning of them hermeneutically. This ongoing and attentive process of interpretation on participants’ interviews allowed the researcher to immerse in the shared understanding on participants’ perspectives and to discover new perspectives that brought the unity of the overall understood meaning.

### 2.2. Researcher’s Reflexivity

The researcher’s own clinical experiences of building therapeutic relationships with older adults experiencing chronic illness catalyzed this qualitative study with the aim to explore other music therapists’ perspectives of this phenomenon. Her strong belief in the importance of QoL and social relationships as being the most significant factors in an individual’s life sparked her curiosity of how this concept of QoL would be perceived by other music therapists. Her assumption was that positive QoL is the primary goal for every client regardless of what that looks like and all music therapists pursue delivering this goal in the interventions. Ultimately, she realized that her own personal perception of QoL affected her approach to constructing music therapy sessions and this deepened her desire to hear from other music therapists who have also been working with older adults with chronic illness. She wanted to know if other therapists had similar perspectives and, if so, what was the significance of this awareness in their relationship-building process within the music therapy session? Would such an awareness change their relationship with older adults or, alternately, does it matter if a therapist has this self-awareness?

### 2.3. Ethical Consideration

Prior to the data collection, approval to conduct this study was granted by the Temple University Institutional Review Board (#25671). After the participants were recruited, the researcher provided them with a consent form via email and asked them to schedule the online interviews at their available time. Pseudonyms were provided for the participants’ confidentiality.

### 2.4. Participants and Setting

A total of 11 female board-certified music therapists who were working with older adults in long-term care were sampled through convenience sampling on social media platforms (Facebook and LinkedIn). In terms of the number of participants, Guest et al. [47] discovered that the data saturation of reflexive thematic analysis was viable just within the first twelve interviews. The researcher recruited ten White female music therapists working in the East Coast, Midwest, and West Coast regions of the United States and one Asian music therapist working in Asia—there was no partiality towards any specific gender. The participants’ age range was from early 20s to mid-60s. All of them were educated and trained in the United States and their levels of education included Ph.D. (one), master’s degree (three), and bachelor’s degree (seven). There were three therapists who have experienced chronic illness (one) and aging (two). To be eligible, the participants had to be board certified music therapists (MT-BC) who were practicing clinicians working with older adults and be able to communicate in English. There were no explicit exclusion criteria that was delineated on the social media post because the study aimed to include diversity, such as stage of life, years of work experience, and cultural backgrounds.

### 2.5. Sampling Procedure and Data Collection

In May 2019, within a two-week period, participants who read the recruiting post on Facebook Forums and LinkedIn Profiles and were interested in participating in the study contacted the researcher via Facebook messages or the researcher’s email address, which was included on the social media post. Then, the researcher sent individual emails to those participants to provide them with more detailed information about the study and the consent form. After receiving the participants’ consent for their participation and their availability of time for an interview, the researcher scheduled each participant’s interview accordingly. In June 2019, semi-structured interviews with nine open-ended questions were conducted using Skype and lasted an average of 30–45 min. Prior to the interview, each participant’s verbal consent was audio-recorded in addition to the whole interview. Afterwards, the audio-recordings were transcribed into text using Microsoft Word with pseudonyms for each participant’s privacy. In this study, the participants are referred to as ‘Participant (PT number)’ instead of pseudonyms for the readers’ clarity.

### 2.6. Data Analysis

This study utilized reflexive thematic analysis to identify meanings and commonalities within and across the data set that were significant in relation to various aspects of the research question [48]. This analysis seemed to be valuable in uncovering the participants’ detailed reflections about the complexity of their lived experiences [49] with older adults experiencing chronic illness. The researcher followed Braun and Clarke’s reflexive thematic analysis, which is: (1) familiarizing yourself with the data, (2) generating initial codes, (3) searching for themes, (4) reviewing themes, (5) defining and naming themes, and (6) producing the report. Initially, these steps were followed in the process of writing the participants’ individual narratives for the purpose of the deeper understanding of their lived experiences based on their interviews. In order to explore these themes, the researcher first uploaded the raw data on ATLAS.ti software [50] and repeated the first and second steps, which created additional codes using open and in vivo coding techniques. Meanwhile, the researcher was submerged in an iterative process of re-reading the transcripts multiple times and identifying recurring words and concepts, which facilitated the creation of preliminary codes. These codes were color-grouped and subsumed into categories, then regrouped and formulated into themes. Additionally, the researcher reworked some categories by clarifying definitions of similar words and eliminating irrelevant and redundant codes and categories. At this stage, using ATLAS.ti, the researcher attempted to create a thematic map that displayed some meaningful overarching themes and this map revealed some profound qualities that underpinned the participants’ clinical and relational approach with their clients. However, in order to engage with the participants’ meaning-bestowing voices and experiences at a more profound level of interpretation, the researcher found that it was necessary to immerse herself in a reflective process of interpretive engagement with the text. This process both included and went beyond the formal analysis of the codes and themes revealed in ATLAS.ti. After this iterative process, the researcher discovered three overarching themes that appeared to highlight the profundity of the participants’ reflection in their engagement with their clients clinically and relationally. These themes are presented in an order that took place within the relationship-building process.

### 2.7. Trustworthiness of the Study

The researcher used member-checking and triangulation as her main method to establish the trustworthiness and credibility of the interpretations and the findings of the study. Prior to the analysis, the researcher asked each participant to verify that the transcript was correct. Triangulation occurred during the analysis process using both software, inductive coding, and reflective discussion with a researcher colleague to compare themes derived from the raw data. This process fostered the researcher’s own reflexive stance and enriched the overall meaning-making process. The profound interconnection among the final themes was supported by the rich and thick descriptions in the findings.

## 3. Results

From the interviews with 11 music therapists, one Asian and ten White, ranging in age from 20 to 60 and educational background from bachelor to Ph.D., the researcher found three important themes that emerged and highlighted the complexity of the participants’ internal perspectives embedded in their relationship-building process with older adults experiencing chronic illness. First, their perspectives underlined two crucial components that must be present in this relationship-building process—the element of music and the quality of the music therapists’ presence. These elements are interdependent in that, together, they facilitate the creation of a meaningful therapeutic environment where older adults can connect and interact with therapists and others emotionally, socially, and musically.

*“We connect through music and the relationship is just deepened. And it’s very pure. We are able to talk through or connect through the music”.*

(PT6)

*“We, as music therapists have a really cool gift to bring to people, because music is a way to connect with those folks and to connect socially, over ideas of, I can play a song and I can tell people based on what I know about their history”.*

(PT9)

As music therapists, all the participants believed that music would provide positive therapeutic experiences and could foster positive relationships with their clients. This was because they used music as their own self-care tool that met their various psychological needs and improved their overall QoL. Additionally, the fact that they were all educated and trained as certified music therapists meant that they were all well-aware of the numerous QoL benefits of music.

*“It’s food for my soul. It is the thing that keeps me balanced; it is the place where I go for refreshment and for energizing”.*

(PT7)

*“Music’s always been a social thing for me. I would walk around the music building, talk to people. I liked learning guitar because I could play the guitar and sing and talk to people”.*

(PT2)

The participants’ positive perceptions of music were predictably embedded in their relationship-building process where they firmly believed that music enhanced the entry point of the therapeutic relationship with intimacy and immediacy.

*“I mean, a big part of it (the relationship building process) too, again, is the music; that instant, therapeutic relationship can be addressed through music”.*

(PT4)

The participants perceived their presence as moderators who created a safe social and psychotherapeutic space for older adults where they could easily express their vulnerability and, in turn, the participants could encounter and connect with older adults’ unique and authentic identity in music.

*“We each have our own music! And everybody’s music is different, but when we hear our music, it really rings TRUE, I think, somewhere within us”.*

(PT9)

Thus, the participants’ relationship-building process was greatly influenced by their positive perceptions of their personal use of music, their knowledge of the psychosocial benefits of music, and their role in facilitating positive therapeutic experiences. The co-existence of music and music therapists was a fundamental pillar to the establishment of the therapeutic relationship and the betterment of older adults’ QoL.

### 3.1. Theme 1: Increased Awareness of Their Unconscious Biases

As the participants entered their relationship-building process, their perspectives on QoL allowed them to experience their increased self-awareness that enabled them to better recognize their unconscious biases and assumptions about the QoL of older adults with chronic illness.

*“…Even though I try to keep that personal stuff, it is what informs what’s going on”.*

(PT1)

*“I’m constantly reminded that, my experience is just not always, it may shade the way I look at things”.*

(PT9)

Since they did not know much about their clients at the beginning stage of the therapeutic process, they could only rely on their past experiences of what they had seen and experienced working with older adults. Although this previous knowledge of older adults’ positive QoL and its components were useful in building rapport, the participants realized that just relying on their previous knowledge was not successful in exploring the individual’s QoL needs and building positive therapeutic relationships that would nurture older adults’ QoL.

*“… My philosophy or my views on quality of life really informed the way I build relationships with people, so putting their interests or what they feel is important to them at the center of… how we’re building that relationship”.*

(PT9)

*“When (I) try to support someone else’s quality of life, it is to make sure that I’m not approaching them just from my own understanding of what quality (of life) means to me”.*

(PT8)

In addition, their increased awareness provided the participants with the ability to identify commonalities with and differences from the QoL values and priorities of older adults with chronic illness. Obtaining this knowledge was imperative because it allowed the participants and older adults to naturally engage with each other at a deeper level of intimacy within the relationship-building process.

*“… Developing or identifying commonalities… through those, you can build a deeper bond”.*

(PT3)

As the participants gained a better self-awareness through their self-reflection, it led them to encounter their own biases and assumptions that influenced their relationship-building process. This realization motivated the participants to modify their biased perceptions of older adults’ QoL and consistently reflect on older adults’ QoL actual needs and priorities rather than acting from their assumptions. This empowered the participants to intentionally seek the common ground they shared with older adults by overlooking their own biases in favor of the unique perspective of each older adult. Thus, the participants’ increased awareness of self and others was a tool that enabled them to identify when they unconsciously projected their own biases.

### 3.2. Theme 2: Deepened Understanding of Older Adults’ QoL

As the participants gained a better awareness of older adults’ QoL, it propelled them to seek a deeper understanding of what a positive QoL really looked like and meant for older adults.

*“It’s about connecting and listening and understanding and understanding includes really those non-verbal things that are not spoken”.*

(PT7)

*“…understanding that something that (you) might think as a QoL might not be exactly right for them”.*

(PT10)

All the participants perceived that the biggest loss of older adults with chronic illness was their loss of independence and the deterioration of their psychosocial and emotional QoL. This often resulted in depression, agitation, and a sense of isolation and abandonment.

*“The biggest quality of life is loss of freedom and their autonomy for them”.*

(PT11)

*“Their loss of independence is out of their control”.*

(PT4)

*“Depression goes along with that (loss of sense of autonomy)”.*

(PT10)

*“They are missing a lot and that might be triggering agitation”.*

(PT11)

*“… (Feelings) that they have are ignored or invalidated”.*

(PT9)

The participants realized that the insurmountable losses of older adults were often incorrectly recognized and misunderstood by others. This could be a result of the outsiders’ sole reliance upon their own perceptions of older adults’ QoL. This highlighted the significance of having a proper understanding of a positive QoL of older adults that can be accomplished by consistently exploring what constitutes a positive QoL for older adults.

*“What is comfort for you (older adults)? Is it spiritual comfort? Do you have physical comfort? I think when someone’s comfortable, that addresses quality of life”.*

(PT4)

The participants also discovered that behind the veil of older adults’ loss of autonomy and independence was an inherent desire to have validation and a sense of meaning in their lives. This realization allowed the participants to find ways to support these desires by developing strong human-to-human connections with older adults and recognizing and respecting their authentic selves.

*“They feel like their place in the world is validated…I’m (older adult) seen as a person, like I’m being recognized in my value, and my dignity is being recognized”.*

(PT9)

*“Living a meaningful life, so have they accomplished things in their life, do they feel like they still have potential to live to their fullest to give back to others?”.*

(PT3)

To support this human-to-human connection, the participants prioritized providing older adults with the unconditional emotional support and trust they needed within the therapeutic relationship.

*“We’re human. So, we’re going to have that human-to-human connection… I think it’s also that trust… trust between your patients and the therapists. I think that is the healthy part of the therapeutic relationship”.*

(PT4)

*“I want to maintain a stance of nonjudgment… (and) being open to whatever experience they are wanting to share”.*

(PT8)

This trustworthy therapeutic relationship required the participants to appropriately use their professional and ethical boundaries and to recognize their clients’ needs from the clients’ perspectives, while remaining aware of their own feelings and responses towards clients. This was a pivotal step that motivated the participants to move from merely having increased awareness of themselves to deepening their understanding of older adults’ QoL.

*“A healthy therapeutic relationship would be (having a) clear boundary… and at the same time, we need to be aware of our own feelings. You see, speaking of countertransference, transference, we DO have to be very, very aware (of) countertransference or transference. (They) are not bad if we manage (them) well… (and) make it therapeutic for the patients”.*

(PT6)

Essentially, the participants’ deepened understanding of older adults’ QoL supported the participants to navigate their relationship-building process with a salutogenic focus. Additionally, this focus entailed providing unconditional empowerment to older adults’ capable and authentic selves that could be fostered through meaningful musical and human-to-human connections. Thus, the participants discovered that it was imperative to constantly seek a better understanding of older adults’ QoL needs and priorities, as older adults have frequent alterations in ability, health, and affect. In doing so, they were able to both apprehend the components of older adults’ positive QoL and reflect on their accountability in building a trustworthy therapeutic relationship with older adults.

### 3.3. Theme 3: Purposeful Alignment with Older Adults’ QoL

The participants’ heightened understanding of older adults’ QoL propelled them to purposefully align with older adults’ QoL. This alignment was a crucial step in not only facilitating meaningful therapeutic experiences for older adults, but also establishing the positive therapeutic relationship for their positive QoL. As the participants purposefully embraced older adults’ unique identity and aligned with their in-the-moment QoL needs, the participants were able to create more emotional intimacy and mutual respect with older adults. This in turn enabled a further development of their trustworthy human-to-human connections with older adults.

*“Being able to shift based on where this person is right now, but also… not carrying the same set of, okay, this is kind of what’s expected in this relationship”.*

(PT8)

*“Building that therapeutic relationship, it’s how can I honor your authentic self… I think there’s this mutual understanding and respect and, of course, that’s going to look different for every person”.*

(PT9)

Additionally, as the participants became more flexible in the pursuit of connecting with older adults’ authentic selves musically and emotionally, they expected themselves to be equally authentic and transparent in the therapeutic relationship. This suggested that the participants’ relationship-building process operated from their essential belief in the equality of human beings.

*“A therapist is not a machine; we are human beings, and if I’m not vulnerable, how am I expecting my patients to be true and vulnerable? So, I also have to be that way, too”.*

(PT6)

*“It’s that general sense of you don’t have to pretend to be a certain person to build a good therapeutic relationship with someone”.*

(PT9)

Thus, the participants could achieve the purposeful alignment with older adults’ QoL only when they were aware of their own biases and understood the older adults’ QoL needs and priorities. Ultimately, when the participants stayed present authentically and connected with older adults intimately on a human-to-human level, the relationship-building process was radically strengthened.

**Figure 1:**
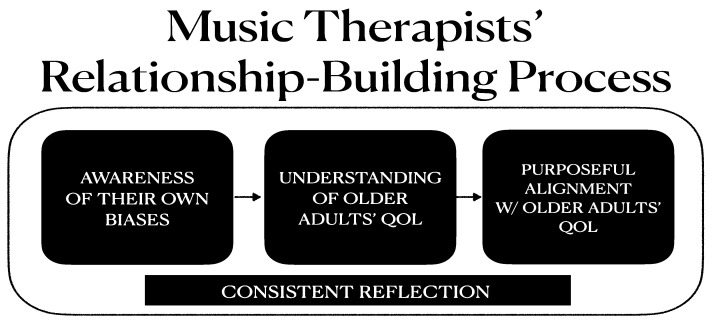
A flow map of music therapists’ perspectives permeated in relationship-building process.

As demonstrated by Figure 1, all three themes revealed the centrality of the participants’ consistent reflection that occurred throughout the relationship-building process with older adults. Most participants appeared to regularly reflect and use their insights to better guide their process of delivering effective clinical interventions as well as building positive therapeutic relationships. The participants’ self-reflection enriched their self-awareness and enhanced their understanding of older adults’ QoL. In doing so, the participants could develop more intimate and meaningful connections with older adults by purposefully focusing on aligning with their in-the-moment QoL needs and conditions. Essentially, the participants’ relationship-building process had to be paired with their reflective process and this was vital to establish the positive therapeutic relationship and to enhance the QoL of older adults with chronic illness.

*“(Self-reflection) helps me have a better understanding about myself and what quality of life means to me”.*

(PT9)

*“Whenever I work with a patient, every session, I have to do a quick self-reflection… It is a quick everyday life to be aware of things that I say, patients’ feelings, my feelings, and how all these values and feelings function in my own system… And when I approach them (older adults) with an awareness, I will do a lot of self-evaluation: What is my approach, is it meeting my needs or patients’ needs?”.*

(PT6)

## 4. Discussion

The results showed that the participants’ reflective process helped them be more intentional in encountering and managing their unconscious biases, which was crucial in not imposing these biases upon older adults’ QoL. The participants recognized that while having their best intentions to help older adults improve their QoL, they were inclined to focus more on older adults’ losses. This medical lens of focusing on problems came from the overreliance on immediate and observable behaviors of older adults. This was the case for eight of the eleven participants who initially focused on QoL differences with their clients based on their perceptions of their clients’ many losses. There were two participants in the study who revealed some significant commonalities in age and chronic health conditions with their older adult clients. One participant, who was of a similar age and had a chronic health condition, thought that not losing relationships was most important for her and older adults’ positive QoL. The second participant who was young but had a chronic health condition found “empathizing with [them] easier” because she resonated with her clients’ physical complications. Since these two participants shared commonalities with their clients, they were not as focused on the older adults’ losses, but rather focused more on how they could empower the older adults’ QoL. Therefore, it is recommended that younger, healthier professionals recognize that working with older adults with chronic illnesses require that they leave behind their bias that loss is the single most important characteristic of older adults. Rather, it is helpful for therapists to reflect upon what an individual older adult truly desires and needs in order to experience a better QoL.

When the participants consciously reflected upon themselves and older adults, they were able to determine what specific unconscious biases were present and how these biases impacted their perceptions of older adults’ in-the-moment needs and conditions. The participants needed to reflect consciously and intentionally to achieve this. Kinsella [51] proposed that reflection is one of the ways that many professionals can foster their knowledge and explore their assumptions about self and others. Since music therapists constantly encounter different people, they face this constant challenge of maintaining alert and sensitive attitudes in order to examine their unconscious biases. In this study, one of the participants who had more knowledge of clinical psychology seemed to be particularly more reflective compared to other participants based on her detailed sharing of her reflective process. This mirrors what Kinsella [51] called “praxis”, a balance of one’s action and reflection within the relationship-building process. Later in the process, this reflection became expanded, and it deepened even more when building trustworthy relationships with older adults. Since having social relationships or contacts was one of older adults’ losses in life that diminished their QoL greatly, the participants could not take this relationship-building process lightly without reflecting upon themselves and on older adults’ distinct QoL components. At this stage, the participants’ persistent reflection (praxis) allowed them to be more sensitive about creating therapeutic boundaries while fostering a humanistic relationship. Reflecting on concepts related to transference and countertransference also helped participants uncover older adults’ unvoiced QoL needs and align themselves and their music experiences with those needs within positive therapeutic relationships.

Good relationships are important to older adults with chronic illness. In this study, the participants strove to build a trustworthy and authentic relationship with the aim to restore and empower older adults’ capable and authentic selves. As the older adults themselves perceived that their connections with others gave them a better QoL [12], the participants recognized that providing older adults with the social environment was crucial to their better QoL. This required the willingness of both the participants and the older adults “to engage in a relationship with an acceptance that vulnerability may arise” [30] (p. 501). Creating trust is essential to building authentic relationships and just as building relationship takes time, so does gaining trust. Thus, it is feasible to suggest that the participants’ reflective process can be useful in both facilitating trust and in understanding and aligning with older adults’ better QoL. Ultimately, this study demonstrated the significance of studying the psychological, social, and health-related aspects of older adults’ QoL [14] and music therapists’ ongoing engagement in their reflection as a mandatory dimension of reflective practitioners. Thus, music therapists’ conscious reflection can prevent their own unconscious biases about older adults’ QoL being imposed in their relationship-building process with older adults experiencing chronic illness.

The majority of the participating music therapists shared commonalities in work settings (elderly long-term and hospice care facilities), clients’ diagnoses (dementias), gender (female), and cultural background (Western). This impeded the exploration of a wider range of clinical settings, chronic illnesses, and participants, which could have affected the findings of this study. Further research might explore the significance of music therapists’ reflective practice and engagement with their clients in relation to their use of music therapy techniques and methods in delivering effective clinical interventions. Additionally, further research might use this relationship-building knowledge and apply it to not only younger and healthier music therapists, but also healthcare professionals in other disciplines who work with older adults with chronic illnesses. This comparison may uncover some interesting factors that could bring out subtle differences in how the therapeutic relationship is established across other disciplines.

## Data Availability

The data are not publicly available due to confidentiality reasons.
